# Influence of Preoperative Handgrip Strength on Length of Stay after Lumbar Fusion Surgery

**DOI:** 10.3390/jcm11143928

**Published:** 2022-07-06

**Authors:** Seo Hee Ko, Sang Jun Park, Na Young Kim, Woohyuk Jeon, Dong Ah Shin, Shin Hyung Kim

**Affiliations:** 1 Department of Anesthesiology and Pain Medicine, Anesthesia and Pain Research Institute, Yonsei University College of Medicine, 50-1 Yonsei-ro, Seodaemun-Gu, Seoul 03722, Korea; shk0207@yuhs.ac (S.H.K.); iotas@yuhs.ac (S.J.P.); knnyyy@yuhs.ac (N.Y.K.); whjeon0804@yuhs.ac (W.J.); 2 Department of Neurosurgery, Yonsei University College of Medicine, 50-1 Yonsei-ro, Seodaemun-Gu, Seoul 03722, Korea

**Keywords:** handgrip strength, sarcopenia, length of stay, lumbar fusion, predictor, outcome

## Abstract

Preoperative sarcopenic status can affect length of hospital stay and patient outcomes after surgery. The aim of this study was to investigate the impacts of preoperative handgrip strength (HGS) on length of stay (LOS) and outcomes after lumbar fusion surgery. HGS was measured preoperatively, and the cut-off value for low HGS was <28 kg for men and <18 kg for women. Perioperative patient outcomes were compared between patients with low and normal HGS. A total of 225 patients, consisting of 86 and 139 patients in the low and normal HGS groups, respectively, fully satisfied the study criteria for analysis. A longer LOS (median 10 vs. 8 days, *p* = 0.013) and a higher incidence of serious postoperative complications (15.1 vs. 3.6%, *p* = 0.002) were observed in the low HGS group. In the multivariate analysis, a low HGS (odds ratio (OR) = 1.917, 95% confidence interval (CI) = 1.046–3.513, *p* = 0.035) was significantly associated with a longer LOS after surgery. Preoperative HGS below the reference values by sex appeared to be an independent factor associated with longer LOS after lumbar fusion surgery.

## 1. Introduction

Handgrip strength (HGS), a measure of voluntary muscle function, has commonly been used as an indicator of overall muscle strength, and measuring HGS is the first step in diagnosing sarcopenia [1,2]. Low HGS is a powerful predictor of poor patient outcomes such as longer hospitalization, increased physical disability, poor health-related quality of life, and mortality [3]. In patients who underwent cardiac surgery, higher mortality rates were observed in patients with low HGS [4]. In addition, preoperative HGS was associated with functional status after hip, knee, and spine surgery [5,6,7].

There has been an increase in lumbar fusion surgery performed worldwide in recent years [8,9]. However, lumbar fusion surgery has been rated as one of the most painful procedures, and significant postoperative risks, including a variety of surgical and medical complications, have been reported [8]. The Enhanced Recovery After Surgery (ERAS) program refers to a multimodal care pathway to accelerate patient recovery after surgery [8]. The most commonly used outcome of the successful implementation of ERAS protocols was the reduction in length of hospital stay (LOS) [8]. Increased LOS has been associated with adverse outcomes and can affect medical costs. In addition, LOS is closely associated with adequate and efficient perioperative management for surgical patients. To date, the effect of only a limited number of factors on LOS has been evaluated for lumbar fusion surgery [10,11]. The high prevalence of sarcopenia has been consistently observed in chronic pain patients with lumbar spinal stenosis [12,13]. However, clinical information on the effect of preoperative sarcopenic status on LOS and on patient outcomes for patients undergoing lumbar fusion surgery is lacking.

The aim of this retrospective observational study was to investigate the impact of preoperative HGS on LOS and outcomes in patients undergoing elective lumbar fusion surgery. In addition, we sought to identify relevant predictors for longer LOS and to evaluate whether low preoperative HGS is independently associated with longer LOS in this population.

## 2. Materials and Methods

### 2.1. Study Population

The study protocol followed the principles of the Declaration of Helsinki and was approved by the Institutional Review Board (no. 4-2021-1598). The requirement for obtaining informed consent from the patients was waived due to the retrospective design of this study. This manuscript adheres to the applicable STROBE guidelines (Supplementary Materials) for observational studies [14]. Patients aged between 20 and 85 years who underwent elective posterior lumbar interbody fusion surgery between November 2016 and March 2018 at a tertiary university hospital were enrolled. In this study, indications for lumbar fusion surgery included the following: spinal stenosis, degenerative spondylolisthesis, herniated intervertebral disc disease, spinal instability, and degenerative scoliosis confirmed by imaging studies. Conservative management was performed for at least 3 months, and surgery was performed if symptoms did not improve. Patients who were able to measure grip strength and had normal cognitive function without diagnosed neurodegenerative disease or psychiatric disorders at the pre-anesthetic visit were included. Patients with a malignancy, an infectious disease, a congenital disease such as cerebral palsy, or trauma, as well as those who underwent multiple lumbar surgeries within the study period, were excluded. Patients with hand osteoarthritis or a neurological disorder that could affect HGS, such as motor neuron disease and Parkinson disease, were excluded.

### 2.2. Handgrip Strength Measure

HGS was measured three times for each hand using a Smedley-type handheld dynamometer (EH101; CAMRY, Guangdong, China) at a pre-anesthetic evaluation visit. We followed the standardized protocol of HGS measurement that has been used in previous studies [4,5,6,7]. HGS was measured and recorded by an independent observer who did not participate in this study. The patients were asked to sit in a comfortable position with their elbows extended to the side and to squeeze the dynamometer with maximum strength. Only one highest value among the three measurements for each hand was recorded and used for the analysis. For the purpose of this study, the patients were divided into two groups—normal HSG (≥28 kg for men and ≥18 kg for women) and low HGS (<28 kg for men and <18 kg for women)—based on the Asian Working Group for Sarcopenia (AWGS) 2019 guidelines [2].

### 2.3. Demographic and Preoperative Clinical Data Measures

Demographic data of age, sex, height, weight, and body mass index (BMI) were collected. Preoperative clinical variables were collected from pre-anesthetic consultation notes and structural medical records. Variables included diagnosed comorbidities that led to continued medical interventions, such as cardiovascular disease, diabetes mellitus (DM), chronic kidney disease, and osteoporosis. Previous lumbar surgery history and the preoperative hemoglobin level were recorded. The Oswestry Disability Index (ODI) for functional status measure was assessed preoperatively [15]. In addition to HGS, muscle mass was estimated by appendicular skeletal muscle mass (ASM) using a previously validated anthropometric equation for the Asian population (ASM (kg) = 0.193 × body weight (kg) + 0.107 × height (cm) − 4.157 × sex (men = 1 or women = 2) − 0.037 × age (years) − 2.631) [16,17]. The skeletal muscle mass index (SMI) was calculated as ASM divided by the square of height in meters.

### 2.4. Intraoperative and Post-Anesthetic Recovery Data Measures

Intraoperative variables such as duration of surgery, estimated blood loss, administration of a transfusion, whether ≥2 levels were fused, and any intraoperative adverse events were investigated. All patients received general anesthesia that consisted of sevoflurane and remifentanil as an intravenous infusion according to the standard protocol. The patient’s condition in the post-anesthesia care unit (PACU) was noted, including the maximum pain scores (numeric rating scale (NRS) of 0 to 10) at the time of admission and discharge, opioid administrations, the Aldrete recovery score (0 to 10) at discharge from PACU [18], and total length of stay in the PACU. In addition, patients who were transferred to the intensive care unit (ICU) because of a need for ventilator care or hemodynamic instability management were identified from the PACU chart.

### 2.5. Postoperative Clinical Data Measures

All of the patients received intravenous patient-controlled analgesia (IV-PCA) after surgery. All of the patients used the same model of disposable PCA pump (Accufuser plus^®^ P2015M; Woo Young Medical, Chungbuk, Korea), which was programmed to deliver 2 mL/h as a basal infusion rate and 0.5 mL per demand, with a 15 min lockout during a 48 h postoperative period. The PCA regimen typically consisted of 10–15 μg/kg of fentanyl with total volume of 100 mL. The next day after surgery, pain scores were measured by a PCA nurse practitioner. The patients were asked the maximum pain scores at rest and at movement for the last 24 h. According to the institutional protocol, patients were allowed to receive rescue opioid analgesics intravenously if they requested additional analgesics or they reported severe pain (≥7 on the NRS), despite IV-PCA use. Hemoglobin level and the transfusion record in the postoperative period were examined. The LOS was defined as the days from surgery to discharge. Serious postoperative complications within 30 days after surgery, such as reoperation due to surgical complications (hematoma or wound infection), delirium, and pneumonia requiring immediate medical interventions, as well as hospital readmission due to uncontrolled pain, were assessed. As long-term surgical outcome-related variables, patient satisfaction (graded on a five-point Likert scale, with 1 = very unsatisfied to 5 = very satisfied) and ODI were assessed at the same follow-up visit between 6 and 12 months after surgery.

### 2.6. Statistical Analysis

Descriptive data were presented as mean ± standard deviation (SD) for continuous variables and as numbers (percentage) for categorical variables. Ordinal data and continuous variables that were not normally distributed were presented as median and interquartile range. The normality of distribution was assessed with the Shapiro–Wilk test. Continuous variables were compared by independent t-test for normal distribution and Mann−Whitney U-test for non-normal distribution. Proportions were analyzed by chi-square test or Fisher’s exact test. Significant univariate variables with a *p*-value threshold of 0.2 were included in the multivariate logistic regression analysis in order to identify the predictors of longer LOS (≥10 days) after lumbar spinal fusion surgery, and the adjusted odds ratio (aOR) and 95% confidence interval (CI) were calculated. Differences in median LOS between the low and normal HGS groups were analyzed using the Kaplan−Meier method and were compared using a log-rank test. All of the statistical analyses were performed using the Statistical Package for the Social Sciences, version 25.0 (IBM Corp, Armonk, NY, USA). A *p*-value <0.05 was considered statistically significant.

## 3. Results

**Figure 1:**
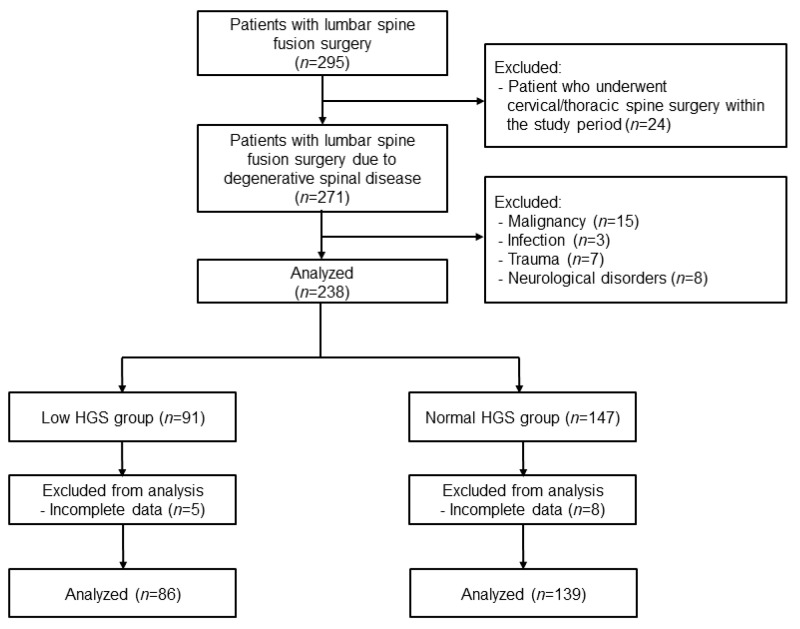
Flow diagram of the study.

A total of 225 patients aged 33 to 84 years, consisting of 139 patients in the normal HGS group and 86 patients in the low HGS group, fully satisfied the study criteria for the analysis (Figure 1).

### 3.1. Demographics and Preoperative Clinical Data Analysis

A comparison of baseline patient demographics and clinical characteristics before surgery between the low and normal HGS groups is listed in Table 1. There were no statistically significant differences in age, sex, and BMI between the two groups. DM was approaching significance in the low HGS group (25.6 vs. 15.1%, *p* = 0.052). Previous lumbar surgery history was more frequently reported in the low HGS group compared with the normal HGS group. The preoperative hemoglobin level was significantly lower in the low HGS group. Poor functional status with a high ODI value was more frequently observed in patients with low HGS. In skeletal muscle mass estimation, patients with a low HGS showed a significantly lower ASM, but there was no significant difference in SMI between the two groups.

### 3.2. Intraoperative and Post-Anesthetic Care Unit Data Analysis

The results of the intraoperative and PACU data analysis are shown in Table 2. The number of patients who received multilevel fusion, the estimated blood loss during surgery, the number of patients who received transfusion, and the total operation time were similar between the two groups. In addition, there were no significant differences between the two groups in pain score, opioid use, recovery score, or total length of stay in the PACU. There was a trend toward significance regarding transfer to the ICU after surgery in the low HSG group (9.3 vs. 2.9%, *p* = 0.063).

### 3.3. Postoperative Course

There was no significant difference in pain intensity or the administration of rescue opioid analgesics between the low and normal HGS groups in the 24 h postoperative period (Table 3). Transfusion after surgery was more frequently required in patients with low preoperative HGS, and it approached the borderline of significance (48.8 vs. 35.9%, *p* = 0.056). A longer LOS (median 10 vs. 8 days, *p* = 0.013) and a higher incidence of serious postoperative complications (15.1 vs. 3.6%, *p* = 0.002) were observed in patients with a low preoperative HGS. There was one case of mortality due to sepsis caused by surgical site infection, which was included in the low HGS group. In long-term follow-up, similar overall surgical outcomes, improved functional status, and favorable patient satisfaction were reported in the two groups.

### 3.4. Factors Associated with Longer Length of Hospital Stay after Surgery

**Figure 2:**
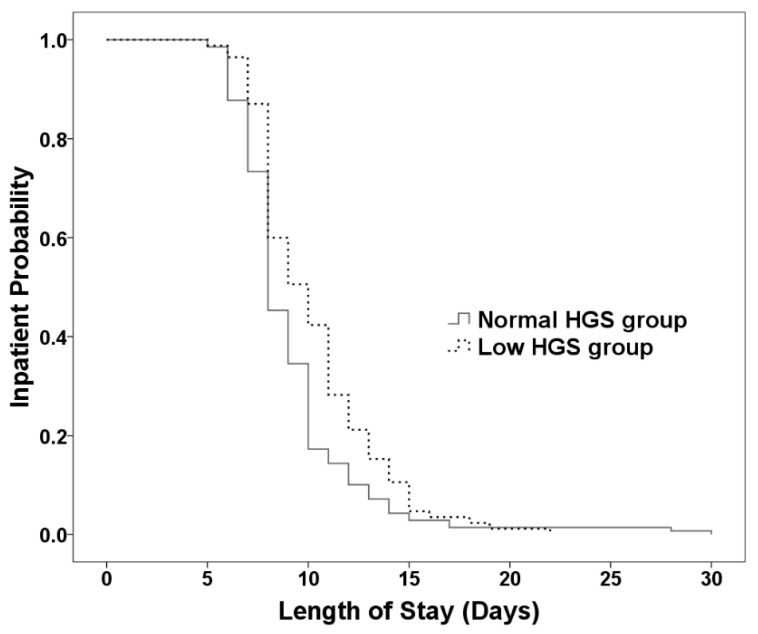
Kaplan−Meier curves for length of hospital stay after lumbar spinal fusion surgery between patients with low and normal handgrip strength (HGS) before surgery (log-rank *p* = 0.006).

Predictive factors among the demographics and perioperative clinical variables for longer LOS (≥10 days) after surgery were identified using univariate and multivariate analyses (Table 4). Among the preoperative factors, low HGS according to the 2019 AWGS guideline (aOR = 1.917, 95% CI = 1.046–3.513, *p* = 0.035) and lower hemoglobin level (aOR = 0.733, 95% CI = 0.598–0.898, *p* = 0.003) were independent predictors for longer LOS after surgery in multivariate analysis. In addition, multilevel fused (aOR = 2.681, 95% CI = 1.412–5.087, *p* = 0.003) and longer duration of surgery (aOR = 1.561, 95% CI = 1.103–2.210, *p* = 0.012) were significantly associated with longer LOS in this population. However, female, DM, lumbar surgery history, ASM, intraoperative blood loss and transfusion, postoperative hemoglobin, transfusion, and complications were not associated with longer LOS after controlling for other variables. The Kaplan−Meier curves for inpatient probability of the low and normal HGS groups are illustrated in Figure 2. The median LOS was significantly longer for patients with low HGS than for those with normal HGS before surgery (10 days [95% CI = 8.809–11.191] vs. 8 days [95% CI = 7.574–8.426], log-rank *p* = 0.006).

## 4. Discussion

In the present study, several different clinical characteristics were observed in patients with preoperative low HGS. Before surgery, the low HGS group showed a higher prevalence of DM. Muscles act as metabolic regulators to maintain specific levels of amino acids and glucose [19]. DM patients with increased muscle strength appeared to have better glycemic control and lower insulin resistance [20]. Preoperative lower hemoglobin levels were observed in the low HGS group. Similar to our results, a recent study reported the hemoglobin level to be independently associated with sarcopenia, and that the association was stronger with muscle strength and function than with muscle mass [21]. History of previous lumbar surgery was more frequently observed in patients with preoperative low HGS. A higher incidence of revisional spinal surgery was reported in patients with sarcopenia because disease in adjacent segments occurred more frequently after surgery [22,23]. In addition, a higher ODI score, indicating poor functional status, was measured in the low HGS group. Low HGS was associated with impaired global skeletal muscle function and disability in daily activities in patients with spinal stenosis [24,25].

In the current study, there was no significant relationship between preoperative HGS and postoperative pain. The elderly population with sarcopenia seemed to have increased susceptibility to the adverse effects of opioid analgesic [26]. Higher pain sensitivity was observed in some subgroups of chronic pain patients with sarcopenia [27]. However, clinical information regarding sarcopenia and acute postoperative pain is lacking. In this study, fentanyl-based patient-controlled analgesia was typically applied postoperatively for all patients. There was no difference in intraoperative parameters or recovery profile between the two groups. A standardized and aggressive pain management protocol for lumbar fusion surgery in our population might affect the results, although some tendency for greater rescue opioid use after surgery was observed in patients with preoperative low HGS.

In this study, we confirmed a higher incidence of postoperative complications in patients with preoperative low HGS. This was consistent with previous studies that showed a high morbidity and mortality in surgical patients with sarcopenia [4,5,6,7,28,29]. In addition, a higher rate of transfusion was reported previously in patients with sarcopenia undergoing repair of femur fractures and thoracolumbar spine surgery [30,31]. Similarly, we observed that more blood transfusions were needed in patients with low HGS in the postoperative period. These factors might contribute to the increased LOS in patients with preoperative low HGS. Although increased LOS after surgery was observed in the low HGS group, long-term surgical outcomes were similar for most patients, regardless of preoperative HGS. McKenzie et al. showed that sarcopenia does not affect long-term clinical outcomes, including functional improvement, after lumbar fusion [32]. In addition, Inose et al. reported that lumbar surgery was equally effective in both sarcopenia and nonsarcopenia patients in terms of pain relief [33].

Some previous reports have focused on predictive factors for long LOS after lumbar surgery, which include old age, poor physical condition, postoperative complications, fusion levels, and postoperative hemoglobin level [10,11,34]. These previous reports did not include any sarcopenia-related clinical parameters. Importantly, our analysis revealed that HGS below the reference values by sex was significantly associated with longer LOS after lumbar fusion surgery. In this study, there was no significant association between estimated muscle mass and LOS. Although bioelectrical impedance analysis or dual-energy X-ray absorptiometry were not used in this study, a recent consensus emphasized muscle strength to be more important than muscle mass as a principle determinant to diagnose sarcopenia [3]. Moreover, sarcopenia, as defined by a reduced skeletal muscle mass, did not impact the clinical success of lumbar surgery or pain intensity and disability in patients with chronic lower back pain [32,33,35]. Thus, measurement of muscle mass alone seemed to have limitations in accurately measuring the quality of muscle and could not reflect the functional strength of muscles.

In our study, a lower preoperative hemoglobin level was strongly associated with increased LOS. Elsamadicy et al. showed that elderly patients with a lower preoperative hemoglobin level had increased LOS and postoperative delirium after spinal fusion [36]. Sanoufa et al. reported perioperative anemia, and the amount of hemoglobin decrease was shown to affect LOS and to increase overall healthcare costs in patients who underwent lumbar surgery [37]. Perioperative anemia increased the frequency of transfusions, and transfusion-related immunomodulation increased the likelihood of postoperative infection and morbidity [38]. In addition, patients with preoperative anemia had a poor oxygen-carrying capacity and thus reduced compensatory physiological mechanisms, resulting in impaired cardiac perfusion, low pulmonary function, and delayed wound healing [39]. Indeed, preoperative anemia assessment and correction are recommended in a recent ERAS guideline for lumbar fusion surgery [8]. Similar to previous reports [10,34], this study confirmed some surgery-related factors, such as that the number of levels fused and the duration of surgery were closely associated with LOS after lumbar fusion surgery. Thus, the results of our study suggest that preoperative HGS and hemoglobin levels are more important for patients requiring multilevel fusion and a longer operation time.

This study had some limitations. Being retrospective in nature, the study could involve selection bias and information bias. In this study, a gait speed test could not be conducted because most of our study population showed neurogenic claudication symptoms. Thus, low HGS alone did not fully satisfy the current diagnosis criteria for sarcopenia. However, a recent study showed that HGS was correlated with walking speed and distance in patients with lumbar spinal stenosis [40]. Patient socioeconomic status, emotional states, and pre-operative medications, which might be related to LOS, could not be investigated in this study. This study used a real-world clinical practice model in which four surgeons decided on the spinal levels fused and surgical techniques, as well as on the postoperative care in the general ward. Although all surgeons had similar clinical experience and followed an institutional guideline for surgical patients, surgeon-related confounders could have played a potential role in determining LOS [11].

## 5. Conclusions

In conclusion, preoperative HGS below the reference values by sex appeared to be an independent factor associated with longer LOS in patients undergoing lumbar fusion surgery. HGS measurement seems to be simple and effective means of assessing the risk for increased LOS and postoperative complications, which could aid in the routine preoperative evaluation of lumbar fusion surgery candidacy. In addition, interventional studies are needed to determine whether muscle strength improvement and anemia correction before surgery can reduce the risk of increased LOS in this population.

## Figures and Tables

**Table 1:** Baseline characteristics of the study subjects.

	Total (*n* = 225)	Low HGS (*n* = 86)	Normal HGS (*n* = 139)	*p*-Value
Patient characteristics				
Age, years	66 ± 9	66 ± 9	65 ± 9	0.314
Female, *n*	134 (59.6)	57 (66.3)	77 (55.4)	0.106
BMI, kg/m^2^	25.0 ± 3.1	25.2 ± 3.3	24.9 ± 3.0	0.387
Comorbid medical disease, *n*				
Cardiovascular disease	110 (48.9)	44 (51.2)	66 (47.5)	0.591
Diabetes mellitus	43 (19.1)	22 (25.6)	21 (15.1)	0.052
Chronic kidney disease	14 (6.2)	7 (8.1)	7 (5.0)	0.349
Osteoporosis	16 (7.1)	7 (8.1)	9 (6.5)	0.637
Preoperative clinical data				
Lumbar surgery history, *n*	60 (26.7)	30 (34.9)	30 (21.6)	0.028
Hemoglobin, g/dL	13.5 ± 1.5	13.2 ± 1.6	13.6 ± 1.4	0.029
ODI, %	53 (35–70)	63 (49–74)	45 (27–59)	0.008
Sarcopenia-related data				
ASM, kg	17.7 ± 4.0	17.0 ± 3.7	18.2 ± 4.2	0.027
SMI, kg/m^2^	6.9 ± 1.0	6.8 ± 1.0	6.9 ± 1.0	0.302
HGS, kg	24.5 ± 10.0	16.8 ± 5.6	29.3 ± 9.1	<0.001

Values are presented as mean ± SD, median (interquartile range), or number of patients (%). *p*-values obtained when comparing the low HGS to the normal HGS group. BMI, body mass index; ODI, Oswestry Disability Index; ASM, appendicular skeletal muscle mass; SMI, skeletal muscle mass index; HGS, handgrip strength.

**Table 2:** Intraoperative and post-anesthetic care unit data.

	Total (*n* = 225)	Low HGS (*n* = 86)	Normal HGS (*n* = 139)	*p*-Value
Intraoperative data				
Multilevel fused, *n*	79 (35.1)	34 (39.5)	45 (32.4)	0.274
Estimated blood loss, mL	600 (400–1000)	600 (400–1000)	600 (400–900)	0.319
Transfusion, *n*	67 (29.8)	31 (36.0)	36 (25.9)	0.106
Duration of surgery, min	196 (160–227)	194 (155–218)	197 (161–236)	0.323
PACU data				
Pain score at admission, 0 to 10	3 (3–3)	3 (3–3)	3 (3–3)	0.299
Use of opioid analgesics, *n*	111 (49.3)	42 (48.8)	69 (49.6)	0.630
Aldrete recovery score at discharge, 0 to 10	10 (10–10)	10 (10–10)	10 (10–10)	0.159
Length of stay in the PACU, min	45 (36–60)	45 (36–61)	45 (35–60)	0.987
Transfer to ICU, *n*	12 (5.3)	8 (9.3)	4 (2.9)	0.063

Values are presented as mean ± SD, median (interquartile range), or number of patients (%). *p*-values obtained when comparing the low HGS to the normal HGS group. PACU, post-anesthetic care unit; HGS, handgrip strength; ICU, intensive care unit.

**Table 3:** Postoperative course.

	Total (*n* = 225)	Low HGS (*n* = 86)	Normal HGS (*n* = 139)	*p*-Value
Pain-related data				
Postoperative—24 h period				
Maximum pain score (rest), 0 to 10	3 (2–5)	3 (2–5)	3 (2–5)	0.656
Maximum pain score (movement), 0 to 10	6 (5–8)	6 (5–8)	7 (5–8)	0.149
Use of rescue opioid analgesics, *n*	106 (47.1)	47 (54.7)	59 (42.4)	0.075
Postoperative clinical data				
Hemoglobin, g/dL	10.7 ± 1.4	10.5 ± 1.5	10.8 ± 1.3	0.116
Transfusion, *n*	92 (40.8)	42 (48.8)	50 (35.9)	0.056
Length of hospital stay, days	9 (8–11)	10 (8–12)	8 (7–10)	0.013
Postoperative complications *, *n*	18 (8.0)	13 (15.1)	5 (3.6)	0.002
Surgical site infection	3 (1.3)	2 (2.3)	1 (0.7)	
Unplanned reoperation	5 (2.2)	3 (3.4)	2 (1.4)	
Pneumonia/ sepsis	5 (2.2)	5 (5.8)	0 (0.0)	
Cardiac arrhythmia	1 (0.4)	1 (1.1)	0 (0.0)	
Delirium	6 (2.6)	5 (5.8)	1 (0.7)	
Readmission	3 (1.3)	1 (1.1)	2 (1.4)	
Mortality	1 (0.4)	1 (1.1)	0 (0.0)	
1-year follow-up data				
ODI, %	28 (16–43)	28 (17–42)	28 (16–43)	0.918
Patient satisfaction **, 1 to 5	4 (4–5)	5 (4–5)	5 (4–5)	0.812

Values are presented as mean ± SD, median (interquartile range), or number of patients (%). *p*-values obtained when comparing the low HGS to the normal HGS group. HGS, handgrip strength; IV-PCA, intravenous patient-controlled analgesia; ODI, Oswestry Disability Index. * Serious postoperative complications requiring immediate surgical or medical intervention within 30 days after surgery. ** A five-point Likert scale with 1 being very unsatisfied and 5 being very satisfied.

**Table 4:** Association of patient demographics and perioperative clinical variables with a longer length of hospital stay (≥ 10 days) after lumbar fusion surgery in univariate and multivariate logistic regression analyses.

	Univariate Analysis			Multivariate Analysis		
	Crude OR	95% CI	*p*-Value	Adjusted OR	95% CI	*p*-Value
Preoperative factors						
Age, per 10 years increase	1.158	0.856–1.566	0.343			
Female, yes	1.751	1.007–3.045	0.047	1.477	0.743–2.935	0.266
BMI, per 1 kg/m^2^ increase	1.048	0.961–1.144	0.289			
Diabetes mellitus, yes	2.118	1.081–4.149	0.029	1.576	0.728–3.410	0.248
Lumbar surgery history, yes	1.661	0.915–3.015	0.095	1.693	0.888–3.227	0.110
Hemoglobin, per 1 g/dL increase	0.758	0.628–0.913	0.004	0.733	0.598–0.898	0.003
ODI, per 1 % increase	0.999	0.975–1.024	0.945			
ASM, per 1 kg increase	0.932	0.871–0.999	0.045	0.980	0.849–1.131	0.785
SMI, per 1 kg/m^2^ increase	0.859	0.660–1.117	0.257			
Low HGS *, yes	1.986	1.147–3.438	0.014	1.917	1.046–3.513	0.035
Intraoperative factors						
Multilevel fused, yes	3.589	2.023–6.369	<0.001	2.681	1.412–5.087	0.003
Estimated blood loss, per 500 mL increase	1.481	1.150–1.907	0.002	1.244	0.867–1.783	0.236
Transfusion, yes	2.115	1.184–3.779	0.011	0.656	0.224–1.922	0.442
Duration of surgery, per 1 h increase	1.660	1.235–2.231	0.001	1.561	1.103–2.210	0.012
Postoperative factors						
Severe pain NRS ≥ 7, yes	0.933	0.515–1.689	0.818			
Rescue opioid use, yes	1.301	0.763–2.219	0.333			
Hemoglobin, per 1 g/dL increase	0.724	0.588–0.891	0.002	1.150	0.842–1.570	0.379
Transfusion, yes	1.956	1.135–3.370	0.016	0.644	0.308–1.346	0.242
Complications, yes	4.127	1.065–15.999	0.040	2.061	0.652–6.519	0.218

OR, odds ratio; CI, confidence interval; BMI, body mass index; ODI, Oswestry Disability Index; ASM, appendicular skeletal muscle mass; SMI, skeletal muscle mass index; HGS, handgrip strength; NRS, numeric rating scale. * Low HGS was defined as <28 kg for men and <18 kg for women according to the Asian Working Group for Sarcopenia 2019 guideline [2].

## Data Availability

Data are available upon request to corresponding author.

## Supplementary Materials

The following supporting information can be downloaded at: https://www.mdpi.com/article/10.3390/jcm11143928/s1, Table S1: STROBE Statement—checklist of items that should be included in reports of observational studies.
